# An Updated Meta-Analysis of Treatment in Patients with Heart Failure Complicated Ventricular Functional Mitral Regurgitation

**DOI:** 10.31083/j.rcm2502048

**Published:** 2024-01-29

**Authors:** Bryan Richard Sasmita, Suxin Luo, Bi Huang

**Affiliations:** ^1^Department of Cardiology, The First Affiliated Hospital of Chongqing Medical University, 400016 Chongqing, China

**Keywords:** functional mitral regurgitation, heart failure, MV repair, mitral annuloplasty, MitraClip

## Abstract

**Background:**

Ventricular functional mitral regurgitation (FMR) is a common morbidity in patients with heart failure (HF). In addition to guideline-directed medical therapy, mitral valve (MV) repair or replacement has become an option for such patients. However, the impact of different treatments on cardiac remodeling, function, and clinical outcomes remains unclear.

**Methods:**

We systematically searched PubMed, EMBASE, Medline, Clinical Trials.gov, and the Cochrane Central Register of Controlled Trials with search terms related to mitral regurgitation, mitral valve repair, surgical mitral valve replacement, mitral annuloplasty device, and MitraClip. The outcomes were left ventricular ejection fraction (LVEF), left ventricular (LV) remodeling, all-cause mortality, cardiovascular death, and HF hospitalization. Sensitivity analysis was performed by removing high-bias risk studies. The analysis was done by Review Manager 5.4 Analyzer and MedCalc Statistical Software version 19.2.6.

**Results:**

This meta-analysis included 10 studies with a total of 2533 patients (567 with transcatheter MitraClip, 823 with surgical MV repair, 651 with surgical MV replacement, and 492 with medical therapy). Our meta-analysis revealed that surgical MV repair had significant improvement in LVEF compared to the surgical MV replacement (mean differences (MD) 2.32, [95% CI 0.39, 4.25]), while transcatheter MitraClip treatment was associated with LVEF reduction (MD –4.82, [95% CI –7.29, –2.34]). In terms of LV remodeling, transcatheter MitraClip treatment was associated with improvement in left ventricular end-diastolic volume (MD –10.36, [95% CI –18.74, –1.99]). Furthermore, compared to surgical MV replacement, surgical MV repair was not associated with a reduction of all-cause mortality (risk ratio (RR) 0.83, [95% CI 0.61, 1.13]) and cardiovascular death (RR 0.95, [95% CI 0.56, 1.62]), while transcatheter MitraClip was associated with reduced risk of all-cause mortality (RR 0.87, [95% CI 0.78, 0.98]).

**Conclusions:**

Surgical MV repair was associated with significant improvement in LVEF but had no significant effect on all-cause mortality compared to surgical MV replacement. Transcatheter MitraClip was associated with better long-term survival than the non-MitraClip group, thus, transcatheter MitraClip could be considered an alternative treatment in patients with HF-complicated ventricular FMR.

## 1. Introduction

Mitral regurgitation (MR) is the most prevalent form of valvular abnormality occurring in up to 10% of the general population [1]. The prevalence of MR increases with age and is often complicated by left ventricular (LV) dysfunction or heart failure (HF) [2]. MR is classified into degenerative MR and functional MR (FMR). The former originates from a structural degeneration of the mitral valve apparatus, while the latter is secondary to LV dysfunction and dilatation due to nonischemic or ischemic causes. Severe systolic dysfunction and LV dilatation often led to ventricular FMR through annular enlargement/dysfunction and leaflet tethering [3]. Such secondary MR increases the severity of hemodynamic strain on the failing LV, contributing to worsening symptoms and low survival [4, 5].

The most common complication of MR is HF or aggravates existing HF. The mortality rate in patients with severe MR is as high as 50% within 5 years, and about 90% of patients experienced at least one hospitalization due to HF [6]. Currently, different therapeutic strategies were recommended based on MR etiologies with mitral valve replacement or repair preferred in degenerative MR and medical therapy as the first-line treatment for FMR [7].

Guideline-directed medical therapy (GDMT) has been proven effective as the mainstay treatment for FMR [7, 8] while the surgical approach remains controversial. Surgical mitral valve replacement has been classified as an IIB indication for patients with severe FMR with New York Heart Association (NYHA) class III–IV [9]. Studies regarding the usefulness of surgical mitral valve (MV) repair and transcatheter MitraClip has been conducted to improve the prognosis of ventricular FMR; however, the comparison between surgical MV repair, surgical MV replacement, transcatheter MitraClip, as well as GDMT reached an inconsistent conclusion [10, 11, 12, 13, 14, 15, 16, 17, 18, 19]. We therefore performed a meta-analysis to compare the outcomes of different treatment methods in patients with HF-complicated moderate to severe ventricular FMR.

## 2. Methods

A systematic literature review was performed in accordance with the Preferred Reporting Items for Meta-Analysis PRISMA Checklist [20]. The methodology was prespecified and published in the International Prospective Register of Systematic Reviews (PROSPERO) (CRD 42023422626).

### 2.1 Search Strategy

We used keywords related to “mitral regurgitation”, “mitral valve repair”, “surgical mitral valve replacement”, “mitral annuloplasty device”, and “MitraClip” searched in PubMed, EMBASE, Medline, Clinical Trials.gov, and The Cochrane Central Register of Controlled Trials databases up to 10 March 2023.

### 2.2 Inclusion and Exclusion Criteria

The inclusion criteria were clinical studies comparing MV repair (surgical MV repair and/or transcatheter MitraClip) and surgical MV replacement or medical therapy in patients with HF-complicated ventricular FMR. Studies without comparison and long-time follow-up data, duplicate publications, articles in a language other than English, and other types of MR were excluded.

### 2.3 Selection and Risk of Bias Assessment

The Cochrane Risk of Bias domains were used to assess the trial eligibility. The selection of domains included sequence generation of allocation, allocation concealment, blinding of outcome assessors, incomplete outcome data, selective outcome reporting, and other sources of bias. Ratings of bias were divided into low risk, unclear risk, and high risk. Studies with high risk or unclear risk of bias for any one of the first three components were considered high-bias risk studies. The quality of evidence was extracted by two independent investigators (BRS and BH), where the third investigator (SXL) will settle the disagreement about the inclusion of data through a discussion and consensus.

### 2.4 Outcomes

Outcomes of interest were (1) Left ventricular function (left ventricular ejection fraction, LVEF); (2) Left ventricular remodeling (left ventricular end-diastolic diameter, LVEDD), left ventricular end-systolic diameter (LVESD), left ventricular end-diastolic volume (LVEDV), and left ventricular end-systolic volume (LVESV); (3) All-cause mortality; (4) Cardiovascular death; and (5) HF-related hospitalization.

### 2.5 Statistical Analysis

Data were analyzed by standard software (Review Manager 5.4 (The Nordic Cochrane Center, The Cochrane Collaboration, Copenhagen, Denmark) and Medcalc 19.2.6 (MedCalc Software bv, Ostend, Belgium)). Outcomes were reported as mean differences (MD) and risk ratios (RRs). Continuous variables were evaluated using MD with standard deviations (SD). Dichotomous data were reported by using Mantel-Haenszel statistical method with 95% confidence intervals (95% CIs). Trials with zero events will not be included in the Analysis. Meta-analysis was performed using both a random-effect model and a fixed-effect model. The effect model was used depending on the degree of heterogeneity ($I^{2}$) and *p*-value. A fixed-effect model was used if $I^{2}$ < 50% and *p*-value > 0.10, while a random-effect model was preferred in high heterogeneity $I^{2}$ > 50% and low *p*-value < 0.10. Sensitivity analysis was performed to identify other sources of heterogeneity, and evaluate the robustness and stability of the outcomes by removing high-bias risk studies.

## 3. Results

### 3.1 Baseline Characteristics

**Fig. 1.**
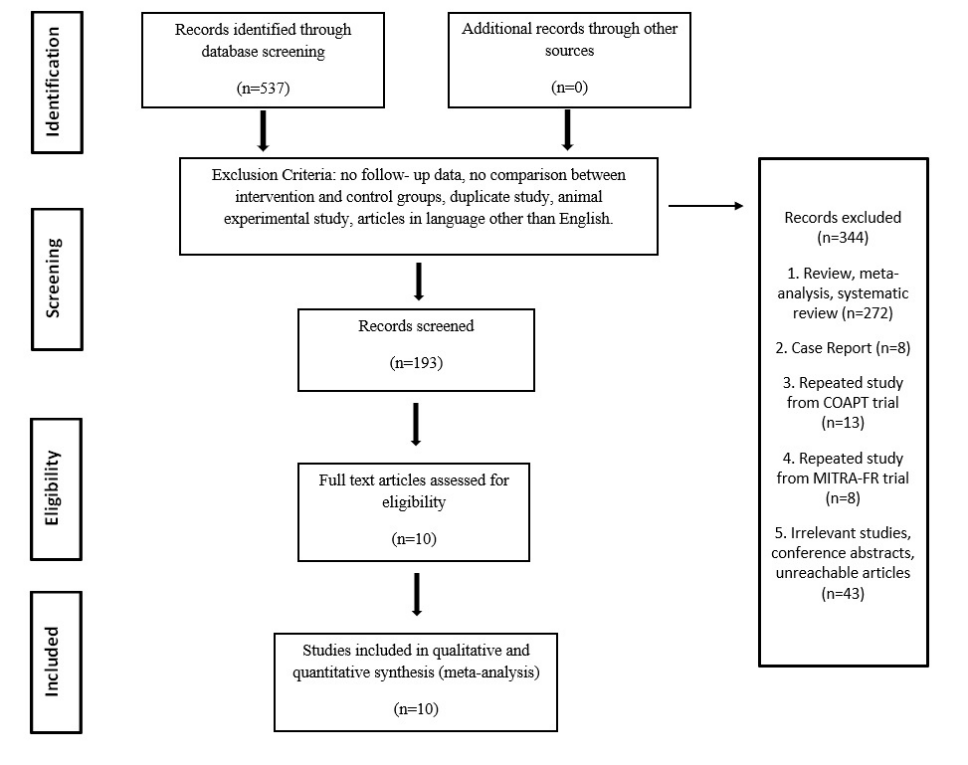
**Flow diagram of data collection**. COAPT, Cardiovascular Outcomes Assessment of the MitraClip Percutaneous Therapy for Heart Failure Patients with Functional Mitral Regurgitation; MITRA-FR, Percutaneous Repair with the MitraClip Device for Severe Functional/Secondary Mitral Regurgitation.

In this meta-analysis, Ten studies with a total of 2533 patients (567 with transcatheter MitraClip, 823 with surgical MV repair, 651 with surgical MV replacement, and 492 with medical therapy) were involved (Fig. 1 and Table 1, Ref. [10, 11, 12, 13, 14, 15, 16, 17, 18, 19]). Compared with the patients who underwent surgical MV replacement, patients who received surgical MV repair tended to have a relatively higher proportion of atrial fibrillation (22.5% vs. 18.42%), less advanced HF (68.7% vs. 71.2%), and higher LVEF (39.17% vs. 38.79%). Moreover, those who underwent transcatheter MitraClip tended to be older (70.53 vs. 69.4 years old), had a higher proportion of atrial fibrillation (43.85% vs. 36.23%), and less advanced HF (67.3% vs. 73.93%) compared to the non-MitraClip group.

**Table 1.** **Select Baseline Characteristics of Included Studies**.

Studies		Calafiore *et al. *2004 [17]	Qiu *et al. *2010 [15]	Bonis* et al*. 2012 [10]	ISTIMIR 2013 [16]	CTSN 2014 [14]	Bonis e*t al. *2015 [11]	MITRA-FR 2018 [12]	Li B e*t al.* 2018 [19]	Papadopoulos *et al. *2020 [18]	COAPT 2023 [13]
***Study Arm***		102	218	132	488	251	120	304	218	86	614
MV Repair		82	112	85	244	126	55	152	109	58	302
Non-MV Repair		20	106	47	244	125	65	152	109	28	312
***Age***											
	MV Repair	66.6 ± 8.3	70.6 ± 8.6	64.3 ± 9.72	66.0 ± 7.1	69 ± 10	68.3 ± 9.17	70.1 ± 10.1	61.72 ± 7.95	72 ± 10	71.7 ± 11.8
	Non-MV Repair	66.2 ± 9.7	71.8 ± 10.8	66.1 ± 8.84	66.1 ± 8.0	68 ± 9	63.2 ± 10.05	70.6 ± 9.9	60.83 ± 8.84	71 ± 11	72.8 ± 10.5
***Male (%)***											
	MV Repair	62 (75.6)	72 (64.3)	62 (72.9)	178 (72.9)	77 (61.1)	46 (83.6)	120 (78.9)	82 (75.2)	42 (72.4)	201 (66.6)
	Non-MV Repair	17 (85.0)	59 (55.7)	36 (76.5)	169 (69.2)	78 (62.4)	45 (69.2)	107 (70.4)	85 (78.0)	25 (86.2)	192 (61.5)
***Atrial Fibrillation (%)***											
	MV Repair	19 (23.2)	31 (27.7)	24 (28.2)	30 (12.2)	45 (35.7)	19 (34.5)	49 (34.5)	8 (7.3)	28 (49.1)	173 (57.3)
	Non-MV Repair	3 (15.0)	28 (26.4)	11 (23.4)	32 (13.1)	35 (28.0)	14 (21.5)	48 (32.7)	5 (4.6)	10 (37.5)	166 (53.2)
***Diabetes (%)***											
	MV Repair	26 (31.7)	33 (29.5)	26 (30.5)	89 (36.4)	48 (38.1)	N/A	50 (32.9)	17 (15.6)	N/A	106 (35.1)
	Non-MV Repair	3 (15.0)	34 (32.1)	14 (29.7)	86 (35.2)	41 (32.8)		39 (25.7)	22 (20.2)		123 (39.4)
***Ischemic Cardiomyopathy (%)***		102 (100)	218 (100)	89 (67.4)	488 (100)	251 (100)	83 (69.2)	180 (59.2)	218 (100)	55 (63.9)	373 (60.7)
***Non-Ischemic Cardiomyopathy (%)***		0	0	43 (32.5)	0	0	37 (30.7)	123 (40.5)	0	31 (36.1)	241 (39.2)
***NYHA Class ≥3 (%)***											
	MV Repair	79 (96.3)	59 (52.7)	58 (68.2)	N/A	72 (57.6)	45 (81.8)	96 (63.1)	N/A	N/A	172 (57)
	Non-MV Repair	20 (100)	52 (49.1)	35 (74.4)		76 (61.3)	56 (86.1)	108 (71.1)			201 (64.6)
***Echocardiography***											
Ejection Fraction (%)											
	MV Repair	38 ± 12	34.6 ± 5.5	30.08 ± 7.7	35.0 ± 3.2	42.4 ± 12.0	27.9 ± 9.84	33.3 ± 6.5	54.94 ± 10.92	31.9 ± 8.4	31.3 ± 9.1
	Non-MV Repair	33 ± 9	35.1 ± 4.3	33.6 ± 7.69	34.9 ± 2.9	40.0 ± 11.0	29.3 ± 6.65	32.9 ± 6.7	56.11 ± 10.06	32.8 ± 6.4	31.3 ± 9.6
LVEDD (mm)											
	MV Repair	N/A	66.29 ± 6.36	66.7 ± 8.77	55.0 ± 7.2	N/A	69.7 ± 7.72	N/A	58.04 ± 6.46	N/A	61.7 ± 7.3
	Non-MV Repair		65.29 ± 6.36	66.1 ± 9.98	55.2 ± 6.9		68.9 ± 6.38		58.43 ± 6.25		61.9 ± 7.5
LVESD (mm)											
	MV Repair	N/A	50.21 ± 11.08	52.7 ± 8.07	42.0 ± 7.0	N/A	54.6 ± 8.81	N/A	N/A	N/A	52.8 ± 8.6
	Non-MV Repair		51.21 ± 11.08	49 ± 13.42	42.2 ± 7.3		52.1 ± 8.21				53.0 ± 8.9
sPAP (mmHg)											
	MV Repair	N/A	47.24 ± 14.31	41.8 ± 12.63	N/A	N/A	47 ± 14.86	N/A	N/A	N/A	44 ± 13.4
	Non-MV Repair		48.01 ± 14.59	46.3 ± 14.12			48.7 ± 13.32				44.6 ± 14.0
***Types of Intervention***											
	MitraClip	-	-	-	-	-	55	152	-	58	302
	Annuloplasty	82	112	85	244	126	65	-	109	-	-
	Surgical Replacement	20	106	47	244	125	-	-	109	-	-
	CABG	93	218	47	488	187	N/A	N/A	185	-	-
	Medical Therapy	-	-	-	-	-	-	152	-	28	312
***Follow-up Time (years)***		3.2	4.1	2.3	3.8	1	4	1	4.9	1	5

MV Repair, surgical MV repair or transcatheter MitraClip; Non-MV Repair, surgical MV replacement or Medical Therapy; MV, mitral valve; LVEDD, left ventricular end-diastolic diameter; LVESD, left ventricular end-systolic diameter; sPAP, systolic pulmonary artery pressure; CABG, coronary artery bypass grafting; COAPT, Cardiovascular Outcomes Assessment of the MitraClip Percutaneous Therapy for Heart Failure Patients with Functional Mitral Regurgitation; MITRA-FR, Percutaneous Repair with the MitraClip Device for Severe Functional/Secondary Mitral Regurgitation; CTSN, the Cardiothoracic Surgical Trials Network; NYHA, New York Heart Association; ISTIMIR, the Italian Study on The Treatment of Ischemic Mitral Regurgitation; N/A, not available.

The high heterogeneity presented in this study may be attributed to an insufficient study number, distinctive measurement index, and different baseline characteristics in each study, such as age, sample size, and follow-up time.

### 3.2 Left Ventricular Function 

**Fig. 2.**
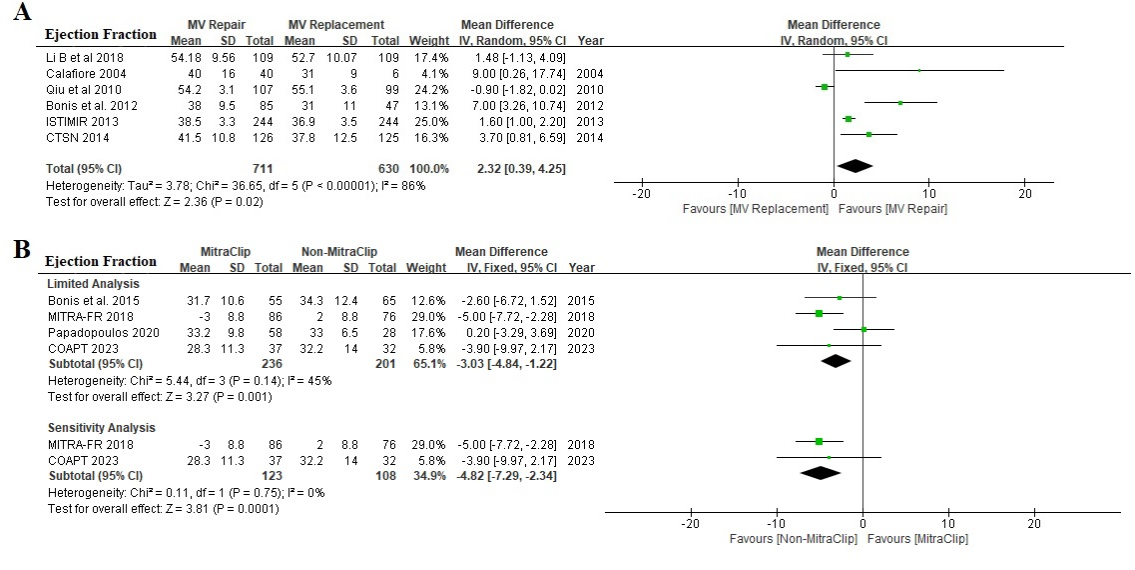
**Effect of MV intervention in left ventricular ejection fraction**. (A) A comparison of the impact of surgical MV repair with surgical MV replacement. (B) A comparison of the impact of transcatheter MitraClip with non-MitraClip (limited analysis and sensitivity analysis). MV, mitral valve; COAPT, Cardiovascular Outcomes Assessment of the MitraClip Percutaneous Therapy for Heart Failure Patients with Functional Mitral Regurgitation; MITRA-FR, Percutaneous Repair with the MitraClip Device for Severe Functional/Secondary Mitral Regurgitation; CTSN, the Cardiothoracic Surgical Trials; ISTIMIR, the Italian Study on The Treatment of Ischemic Mitral Regurgitation.

Ten clinical studies with 1778 patients (711 in surgical MV repair, 630 in surgical MV replacement, 236 in transcatheter MitraClip, and 201 in medical therapy) were analyzed. The median follow-up was 3 years. The randomized effect model limited analysis showed that patients who received surgical MV repair had significant improvement in LVEF compared with those who received surgical MV replacement (MD 2.32, [95% CI 0.39, 4.25], $I^{2}$ 86%) (Fig. 2A). The fixed effect model limited analysis and sensitivity analysis was done in patients who underwent transcatheter MitraClip and demonstrated that the transcatheter MitraClip was associated with reduced LVEF (MD –3.03, [95% CI –4.84, –1.22], $I^{2}$ 45% and MD –4.82, [95% CI –7.29, –2.34], $I^{2}$ 0%, respectively) (Fig. 2B).

### 3.3 Left Ventricular Remodeling

**Fig. 3.**
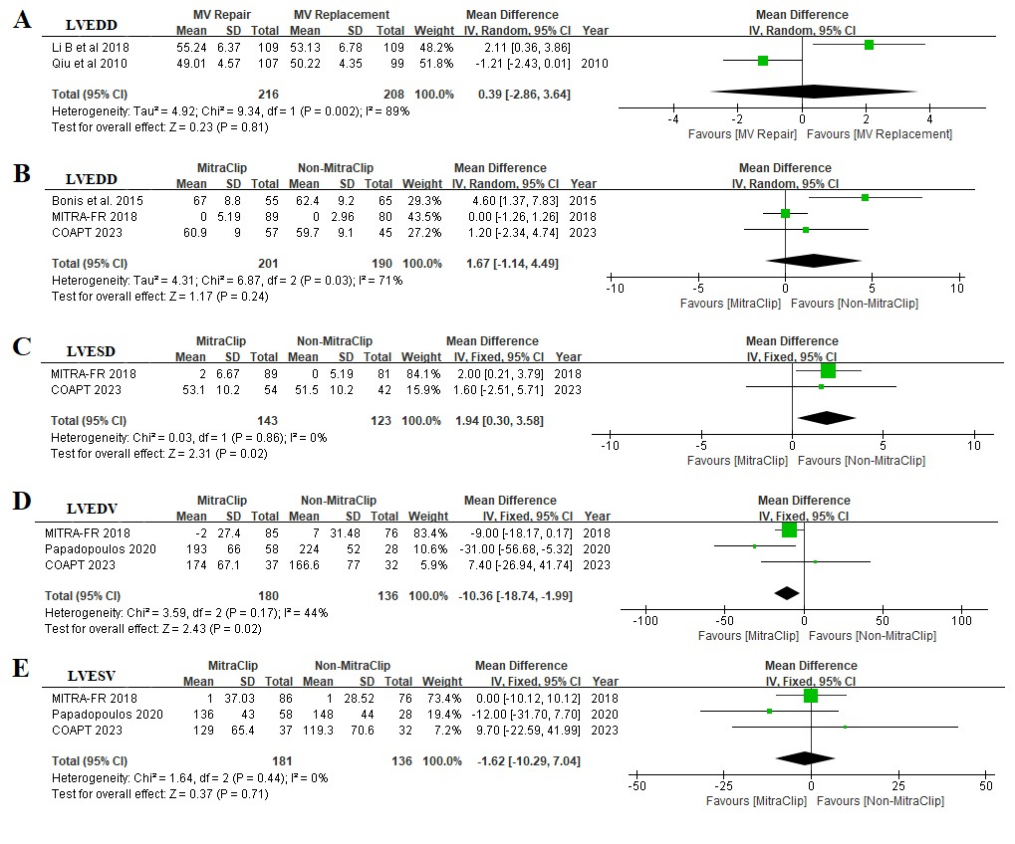
**Effect of MV intervention in LV Remodeling**. (A) A comparison of the impact of surgical MV repair and surgical MV replacement in LVEDD. (B) A comparison of the impact of transcatheter MitraClip and Non-MitraClip in LVEDD. (C) A comparison of the impact of transcatheter MitraClip and Non-MitraClip in LVESD. (D) A comparison of the impact of transcatheter MitraClip and Non-MitraClip in LVEDV. (E) A comparison of the impact of transcatheter MitraClip and Non-MitraClip in LVESV. MV, mitral valve; LV, left ventricular; LVEDD, left ventricular end-diastolic diameter; LVESD, left ventricular end-systolic diameter; LVEDV, left ventricular end-diastolic volume; LVESV, left ventricular end-systolic volume; COAPT, Cardiovascular Outcomes Assessment of the MitraClip Percutaneous Therapy for Heart Failure Patients with Functional Mitral Regurgitation; MITRA-FR, Percutaneous Repair with the MitraClip Device for Severe Functional/Secondary Mitral Regurgitation.

Fig. 3 shows the impact of MV intervention in left ventricular remodeling in Six studies (259 in transcatheter MitraClip, 218 in medical therapy, 216 in surgical MV repair, and 208 in surgical MV replacement). Limited analysis showed that surgical MV repair was not associated with improvement of LVEDD (MD 0.39, [95% CI –2.86, 3.64], $I^{2}$ 89%). In contrast, transcatheter MitraClip was associated with improvement in LVEDV (MD –10.36, [95% CI –18.74, –1.99], $I^{2}$ 44%), but not in LVEDD (MD 1.67, [95% CI –1.14, 4.49], $I^{2}$ 71%) or LVESV (MD –1.62, [95% CI –10.29, 7.04], $I^{2}$ 0%). In addition, a remarkable improvement of LVESD was observed in the Non-MitraClip group (MD 1.94, [95% CI 0.30, 3.58], $I^{2}$ 0%).

### 3.4 All-Cause Mortality

**Fig. 4.**
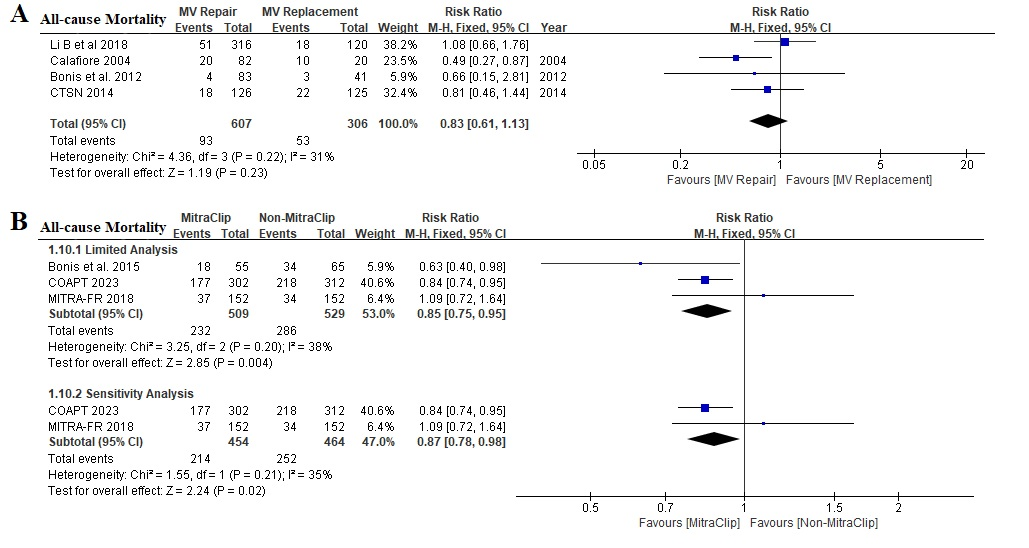
**Effect of MV intervention on all-cause mortality**. (A) A comparison of the impact of surgical MV repair and surgical MV replacement on all-cause mortality. (B) A comparison of the impact of transcatheter MitraClip and Non-MitraClip on all-cause mortality (limited analysis and sensitivity analysis). MV, mitral valve; CTSN, the Cardiothoracic Surgical Trials Network; COAPT, Cardiovascular Outcomes Assessment of the MitraClip Percutaneous Therapy for Heart Failure Patients with Functional Mitral Regurgitation; MITRA-FR, Percutaneous Repair with the MitraClip Device for Severe Functional/Secondary Mitral Regurgitation.

Three randomized controlled trials and Four retrospective studies evaluated the effect of MV repair on all-cause mortality (Fig. 4). Limited analysis showed that transcatheter MitraClip was associated with a lower risk of all-cause mortality (RR 0.85, [95% CI 0.75, 0.95], $I^{2}$ 38%), but not in surgical MV repair (RR 0.83, [95% CI 0.61, 1.13], $I^{2}$ 31%). Sensitivity analysis with removing high-bias risk studies showed a consistent result in patients with transcatheter MitraClip (RR 0.87, [95% CI 0.78, 0.98], $I^{2}$ 35%).

### 3.5 HF Re-Hospitalization

**Fig. 5.**
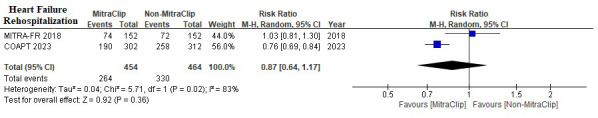
**A comparison of the impact of transcatheter MitraClip and Non-MitraClip on HF-related hospitalization**. HF, heart failure; COAPT, Cardiovascular Outcomes Assessment of the MitraClip Percutaneous Therapy for Heart Failure Patients with Functional Mitral Regurgitation; MITRA-FR, Percutaneous Repair with the MitraClip Device for Severe Functional/Secondary Mitral Regurgitation.

HF-related hospitalization was reported in Two studies. As is shown in Fig. 5, HF re-hospitalization was significantly higher in the Non-MitraClip group (71.12% vs. 58.15%), however, in the limited analysis, transcatheter MitraClip was not associated with improvement of HF re-hospitalization (RR 0.87, [95% CI 0.64, 1.17], $I^{2}$ 83%).

### 3.6 Cardiovascular Death

**Fig. 6.**
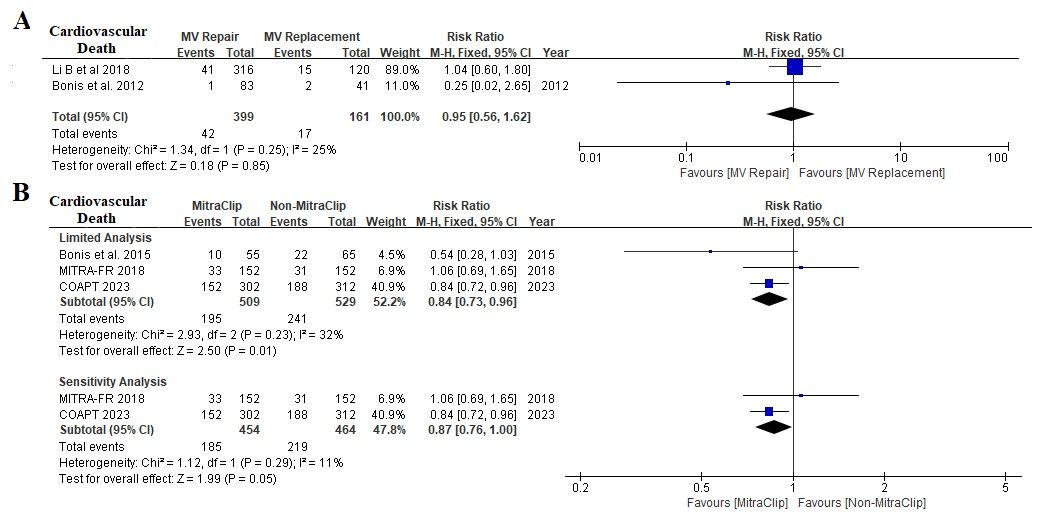
**Effect of MV Intervention on cardiovascular death**. (A) A comparison of the impact of surgical MV repair and surgical MV replacement on cardiovascular death. (B) A comparison of the impact of transcatheter MitraClip with Non-MitraClip on cardiovascular death (limited analysis and sensitivity analysis). MV, mitral valve; COAPT, Cardiovascular Outcomes Assessment of the MitraClip Percutaneous Therapy for Heart Failure Patients with Functional Mitral Regurgitation; MITRA-FR, Percutaneous Repair with the MitraClip Device for Severe Functional/Secondary Mitral Regurgitation.

Two randomized clinical trials and three observational studies evaluated the effect of MV intervention on cardiovascular death (Fig. 6). Limited analysis showed that transcatheter MitraClip was associated with a reduction of cardiovascular death (RR 0.84; [95% CI 0.73, 0.96], $I^{2}$ 25%), but not in surgical MV repair (RR 0.95, [95% CI 0.56, 1.62], $I^{2}$ 25%). However, sensitivity analysis revealed this association was marginally significant (RR 0.87, [95% CI 0.76, 1.00], $I^{2}$ 11%).

### 3.7 Risk of Bias and Quality Assessment

**Fig. 7.**
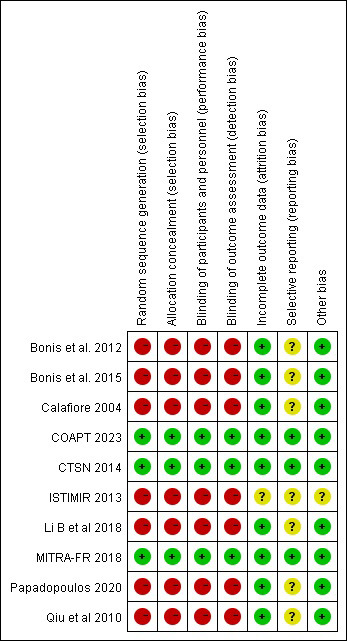
**Risk of Bias**.

Based on the Cochrane Collaboration for risk of bias assessment criteria, enrolled studies presented with various risks of bias (Fig. 7). Moreover, the assessment of other possible biases is uncertain due to insufficient information from respective studies.

## 4. Discussion

In the present meta-analysis, we compared the impact of different treatments of MV on cardiac remodeling and function in patients with HF-complicated ventricular FMR. Although surgical MV repair was associated with improved cardiac function, it was not associated with better survival. In contrast, the transcatheter MitraClip was superior in reducing all-cause mortality but not associated with LV function improvement in patients with HF-complicated ventricular FMR.

HF is a chronic clinical syndrome induced by structural or functional cardiac abnormalities [21]. With the progression of the disease over time, ventricular FMR may occur as a consequence of LV remodeling and systolic dysfunction. Etiologies of FMR are subclassified as ischemic and non-ischemic. Ischemic mitral regurgitation is the most common type of FMR, with acute myocardial infarction leading to LV remodeling as the main mechanism. On the other hand, non-ischemic mitral regurgitation is often presented in patients with dilated cardiomyopathy and atrial fibrillation. Ventricular FMR is characterized by apical and posterior displacement of papillary muscles, leaflet tethering, and incomplete systolic MV closure caused by LV dysfunction and remodeling [3]. Enlarged left atrial and ventricular causes the mitral annulus to dilate and lose its saddle shape, resulting in increased MV leaflet area, increased leaflet stress, and eventually failure of coaptation [22, 23]. Moreover, FMR will gradually exaggerate LV remodeling by increasing the volume load. This complex relationship not only contributes to the disease’s progression and severity but is also associated with poor prognosis [24, 25].

GDMT is the first-line treatment for patients with HF-complicated ventricular FMR [7]. Meanwhile, cardiac resynchronization therapy has also been shown to be effective in treating FMR by restoring synchronous ventricular contraction in patients with complete left bundle branch block [26]. Despite advances in medical and mechanical therapy, the prognosis of these patients remains poor [6, 27]. Mitral valve annuloplasty (MVA) or surgical MV repair is an option for patients with HF-complicated ventricular FMR. However, Wu *et al*. [28] found that MVA did not significantly influence mortality in patients with significant MR and severe LV dysfunction. Ischemic cardiomyopathy is the most common cause of FMR. In a study with 390 ischemic MR patients, 290 received coronary artery bypass grafting (CABG) with MVA, and 100 received CABG alone [29]. It was found that CABG with MVA improved early symptoms but had no significant improvement in long-term functional status or survival [29]. Therefore, MVA seems to have no significant influence on mortality in patients with HF-complicated ventricular FMR. Based on the present updated meta-analysis, our findings were consistent with previous findings [12, 13, 26, 29], which indicated that although surgical MV repair could improve cardiac function, it could not reduce the risk of mortality.

MV replacement has been preferred in patients with severe secondary MR [9], however, despite these recommendations, the evidence remains low. The Cardiothoracic Surgical Trials Network (CTSN) [15] compared surgical MV repair with chordal-sparring replacement in patients with severe ischemic MR and found no significant difference in survival or LV reverse remodeling. In the present meta-analysis, we compared surgical MV replacement with surgical MV repair and found that surgical MV repair was associated with better LVEF improvement (MD 2.32, [95% CI 0.39, 4.25]) but had no significant impact on all-cause mortality (RR 0.83, [95% CI 0.61, 1.13]). Previous studies have shown that LVEF worsened after MV replacement [14, 16]; however, the exact mechanisms were still unclear. A possible interpretation is the restoration of the normal LV geometric relationship, as well as progressive positive LV remodeling, which allows a decrease in LV end-systolic volume and therefore leads to an improvement in LV stroke volume and LVEF [30]. However, more studies are still needed to clarify the precise mechanism.

Percutaneous therapy or trans-catheter MV repair, especially MitraClip, has recently gained much attention. Transcatheter MitraClip has become a preferred treatment choice in patients with severe FMR due to its safety, high procedural success rate, and its ability to improve hemodynamic and functional status [31, 32]. Although transcatheter MitraClip recently has been classified as a class IIA indication for severe secondary MR patients with NYHA class III-IV HF symptoms, [33] several randomized clinical trials reported conflicting results. EVEREST II (Endovascular Valve Edge-to-Edge Repair Study) [34] and MITRA-FR (Percutaneous Repair with the MitraClip Device for Severe Functional/Secondary Mitral Regurgitation.) [12] demonstrated no clinical benefit after the correction of FMR with MitraClip, while the COAPT trial (Cardiovascular Outcomes Assessment of the MitraClip Percutaneous Therapy for Heart Failure Patients with Functional Mitral Regurgitation) [13] with 5-year follow-up demonstrated that treating FMR with MitraClip was associated with a lower rate of all-cause mortality and hospitalization due to HF. The conflicting results were interpreted possibly by the different clinical characteristics among the studies. For example, the inclusion criteria of severe MR between the MITRA-FR and COAPT trials differed. The former was based on European guidelines (effective regurgitant orifice area (EROA) >20 ${\mathrm{mm}}^{2}$ or regurgitant volume (RV) >30 mL), while the latter was based on more strict American guidelines (EROA >30 ${\mathrm{mm}}^{2}$ or RV >45 mL), which resulted in a larger EROA in COAPT trial (41 ± 15 vs. 31 ± 10 ${\mathrm{mm}}^{2}$). In the present analysis, we found that transcatheter MitraClip was associated with a lower risk of all-cause mortality and LVEF decrease. In fact, reduced low-impedance atrial leak and increased forward stroke volume have been recognized as key mechanisms contributing to a reduction in LVEF after transcatheter MitraClip implantation. Although the present study found that transcatheter MitraClip was not associated with LVEF improvement, however, transcatheter MitraClip implantation in strictly screened patients could reduces the risk of all-cause mortality and hospitalization due to HF. Therefore, patient selection is critical for transcatheter MitraClip implantation.

## 5. Limitations

Several limitations should be addressed in this study. First, the present meta-analysis included three randomized controlled trials and seven observational studies to evaluate the efficacy of MV repair in patients with HF-complicated ventricular FMR. The high heterogeneity and higher risk of selection bias from observational studies may affect the reliability of the present meta-analysis. Therefore, careful interpretation is needed. Second, echocardiographic indexes are easily affected by afterload and preload, thus careful and repeated measurement is necessary. Third, a wide range of variables exist, such as small sample size, different outcomes, different GDMT regimens, as well as concomitant procedures limiting the statistical power and preferred outcomes, thus, careful interpretation and more large-scale studies are needed to clarify the weight of MV intervention in patients with HF-complicated ventricular FMR.

## 6. Conclusions

Surgical MV repair was associated with significant improvement in LVEF but had no significant effect on all-cause mortality compared to surgical MV replacement. Transcatheter MitraClip was associated with better long-term survival than the non-MitraClip group, thus, transcatheter MitraClip could be considered an alternative treatment in patients with HF-complicated ventricular FMR.

## Data Availability

All data relevant to the study are included in the article or uploaded as supplementary files. Data can also be requested from the corresponding author.

## Supplementary Material

Supplementary material associated with this article can be found, in the online version, at https://doi.org/10.31083/j.rcm2502048.
