# Supplementary material for: An Updated Meta-Analysis of Treatment in Patients with Heart Failure Complicated Ventricular Functional Mitral Regurgitation
Source: Rev Cardiovasc Med. 2024 Jan 29;25(2):48. doi: 10.31083/j.rcm2502048 (PMC11263179; doi:10.31083/j.rcm2502048)
Supplement: Supplementary file 1 [file 2153-8174-25-2-048-s1.zip › 2153-8174-25-2-048-s1/CRD42023422626.pdf]

National Institute for Health Research

UNIVERSITY of York
Centre for Reviews and Dissemination

# Systematic review

A list of fields that can be edited in an update can be found here

## 1. * Review title.

Give the title of the review in English

An updated meta-analysis of treatment in patients with Heart Failure complicated Functional Mitral Regurgitation

## 2. Original language title.

For reviews in languages other than English, give the title in the original language. This will be displayed with the English language title.

English

## 3. * Anticipated or actual start date.

Give the date the systematic review started or is expected to start.

01/03/2023

## 4. * Anticipated completion date.

Give the date by which the review is expected to be completed.

25/06/2023

## 5. * Stage of review at time of this submission.

**This field uses answers to initial screening questions. It cannot be edited until after registration.**

Tick the boxes to show which review tasks have been started and which have been completed.

Update this field each time any amendments are made to a published record.

The review has not yet started: No

| Review stage | Started | Completed |
|---|---|---|
| Preliminary searches | Yes | No |
| Piloting of the study selection process | Yes | No |
| Formal screening of search results against eligibility criteria | No | No |
| Data extraction | No | No |
| Risk of bias (quality) assessment | No | No |
| Data analysis | No | No |

Provide any other relevant information about the stage of the review here.

## 6. * Named contact.

The named contact is the guarantor for the accuracy of the information in the register record. This may be any member of the review team.

Bryan Richard Sasmita

## Email salutation (e.g. "Dr Smith" or "Joanne") for correspondence:

Dr Bryan

## 7. * Named contact email.

Give the electronic email address of the named contact.

1400885752@qq.com

## 8. Named contact address

Give the full institutional/organisational postal address for the named contact.

40016

## 9. Named contact phone number.

Give the telephone number for the named contact, including international dialling code.

15683404416

## 10. * Organisational affiliation of the review.

Full title of the organisational affiliations for this review and website address if available. This field may be completed as 'None' if the review is not affiliated to any organisation.

The First Affiliated Hospital of Chongqing Medical University

## Organisation web address:

## 11. * Review team members and their organisational affiliations.

Give the personal details and the organisational affiliations of each member of the review team. Affiliation refers to groups or organisations to which review team members belong. **NOTE: email and country now MUST be entered for each person, unless you are amending a published record.**

Dr Bryan Richard. The First Affiliated Hospital of Chongqing Medical University
Professor Suxin Luo. The First Affiliated Hospital of Chongqing Medical University
Professor Bi Huang. The First Affiliated Hospital of Chongqing Medical University

## 12. * Funding sources/sponsors.

Details of the individuals, organizations, groups, companies or other legal entities who have funded or sponsored the review.

None

## Grant number(s)

State the funder, grant or award number and the date of award

## 13. * Conflicts of interest.

List actual or perceived conflicts of interest (financial or academic).

None

## 14. Collaborators.

Give the name and affiliation of any individuals or organisations who are working on the review but who are not listed as review team members. **NOTE: email and country must be completed for each person, unless you are amending a published record.**

## 15. * Review question.

State the review question(s) clearly and precisely. It may be appropriate to break very broad questions down into a series of related more specific questions. Questions may be framed or refined using PI(E)COS or similar where relevant.

Is mitral valve repair better than guideline-directed medical therapy or mitral valve replacement in patients with severe functional mitral regurgitation?

## 16. * Searches.

State the sources that will be searched (e.g. Medline). Give the search dates, and any restrictions (e.g. language or publication date). Do NOT enter the full search strategy (it may be provided as a link or attachment below.)

Studies for this review and meta-analysis will be accessed through electronic or non-electronic and other relevant sources. We intend to use common database searches, such as PubMed, EMBASE, MEDLINE, Clinical Trials.gov, and The Cochrane Central Register of Controlled Trials. Our search will include all published studies written in English language through the final search date of 10 March 2023. The searches will be conducted using MeSH terms, combined key terms, text words, and search strings taken from the review questions. To access the records, the keywords related to mitral regurgitation, mitral valve repair, surgical mitral valve replacement, mitral annuloplasty device, and MitraClip will be used. After identifying key relevant articles, their references will also be looked into (ancestor search strategy). Similarly, other studies which cited them will be looked on the line (descendent search strategy).

## 17. URL to search strategy.

Upload a file with your search strategy, or an example of a search strategy for a specific database, (including the keywords) in pdf or word format. In doing so you are consenting to the file being made publicly accessible. Or provide a URL or link to the strategy. Do NOT provide links to your search **results**.

Alternatively, upload your search strategy to CRD in pdf format. Please note that by doing so you are consenting to the file being made publicly accessible.

Do not make this file publicly available until the review is complete

## 18. * Condition or domain being studied.

Give a short description of the disease, condition or healthcare domain being studied in your systematic review.

Impact of mitral valve repair compared to guideline-directed medical therapy and mitral valve replacement in patients with heart failure complicated functional mitral regurgitation.

## 19. * Participants/population.

Specify the participants or populations being studied in the review. The preferred format includes details of both inclusion and exclusion criteria.

Heart failure complicated functional mitral regurgitation

## 20. * Intervention(s), exposure(s).

Give full and clear descriptions or definitions of the interventions or the exposures to be reviewed. The preferred format includes details of both inclusion and exclusion criteria.

Mitral valve repair

## 21. * Comparator(s)/control.

Where relevant, give details of the alternatives against which the intervention/exposure will be compared (e.g. another intervention or a non-exposed control group). The preferred format includes details of both

inclusion and exclusion criteria.

Mitral valve replacement and guideline-directed medical therapy

## 22. * Types of study to be included.

Give details of the study designs (e.g. RCT) that are eligible for inclusion in the review. The preferred format includes both inclusion and exclusion criteria. If there are no restrictions on the types of study, this should be stated.

All types of published comparative studies between mitral valve repair with mitral valve replacement of guideline-directed medical therapy in patients with heart failure complicated functional mitral regurgitation will be included (cross-sectional, cohort, case-control, and randomized controlled trials).

## 23. Context.

Give summary details of the setting or other relevant characteristics, which help define the inclusion or exclusion criteria.

We will contain studies that have been conducted in either a community or facility setting, and which involve an analysis of primary or secondary data.

## 24. * Main outcome(s).

Give the pre-specified main (most important) outcomes of the review, including details of how the outcome is defined and measured and when these measurement are made, if these are part of the review inclusion criteria.

The effect of mitral valve repair on cardiac function and remodeling in patients with heart failure complicated functional mitral regurgitation

### Measures of effect

Please specify the effect measure(s) for you main outcome(s) e.g. relative risks, odds ratios, risk difference, and/or 'number needed to treat.

Mean differences and relative risks

## 25. * Additional outcome(s).

List the pre-specified additional outcomes of the review, with a similar level of detail to that required for main outcomes. Where there are no additional outcomes please state ‘None’ or ‘Not applicable’ as appropriate to the review

Assessment of MitraClip in patients with heart failure complicated functional mitral regurgitation.

### Measures of effect

Please specify the effect measure(s) for you additional outcome(s) e.g. relative risks, odds ratios, risk difference, and/or 'number needed to treat.

Mean differences and relative risks

## 26. * Data extraction (selection and coding).

Describe how studies will be selected for inclusion. State what data will be extracted or obtained. State how this will be done and recorded.

All searches of the electronic databases will be conducted using MeSH terms, keywords, text words, and search strings appropriate to each database. The selection process will be started by carefully reading the title, followed by the abstract, the objectives, and the full body of each article and paper. Each paper will be evaluated in accordance with PRISMA (2020) guidelines. The titles of the papers and articles that will be clearly mentioned the relationship between mitral valve repair and improvement of cardiac function in patients with heart failure complicated mitral regurgitation will be identified to be evaluated by the checklist. Articles identified as potentially relevant based on their titles will be further evaluated by reading their full text, including reference to objectives, methods/approaches, and participants/populations and results. Studies that are not eligible based on the examination of the full text will be excluded, and the reasons for the exclusion will be documented. A study selection process flow diagram will be presented based on the PRISMA statement and other relevant literature. Any disagreements arising over inclusion will be addressed, and the third investigator will settle the dispute through a discussion and consensus. After the selection of appropriate articles, the findings from the articles will be extracted using a data extraction template (Microsoft Word 2010). The quantitative data (the number of participants with and without outcome), and the total sample size (n) will be extracted from each article and summarized using Microsoft Excel spreadsheet for systematic review and meta-analysis, and the data will be presented using Microsoft Word template. The overall selected search results will be exported and compiled by using EndNote citation manager software. Articles in which the exposure and outcome variables are not clearly indicated, studies that do not use appropriate sample size determination or sampling methods will be excluded.

## 27. * Risk of bias (quality) assessment.

State which characteristics of the studies will be assessed and/or any formal risk of bias/quality assessment tools that will be used.

The Cochrane Risk of Bias domains were used to assess the trial eligibility. The selection of domains included sequence generation of allocation, allocation concealment, blinding of outcome assessors, incomplete outcome data, selective outcome reporting, and other sources of bias. Ratings of bias were divided into low risk, unclear risk, and high risk. Studies with high risk or unclear risk of bias for any one of the first three components were considered high-bias risk studies. Quality of evidence extracted by two independent investigators, where the third investigator will settle the disagreement about the inclusion of data through a discussion and consensus.

## 28. * Strategy for data synthesis.

Describe the methods you plan to use to synthesise data. This **must not be generic text** but should be **specific to your review** and describe how the proposed approach will be applied to your data. If meta-

analysis is planned, describe the models to be used, methods to explore statistical heterogeneity, and software package to be used.

Data synthesis and statistical analysis will be conducted using Review Manager (RevMan) version 5.4 and MedCalc version 19.2.6. A meta-analysis of observational studies will be carried out based on the recommendations of the $I^2$ statistic described by Higgins et al. (an $I^2$ of 75/100% suggesting considerable heterogeneity). Publication bias will be checked="checked" value="1" using funnel plot asymmetry and the statistical significance test by Egger's linear correlation. If the test results indicate the possible existence of significant publication bias (p=0.01 in Egger's test), the final effect size will be determined by applying trim and fill analysis. A meta-analysis to compute a pooled estimate will be performed if the variability among the studies is low. However, if pooling of the data is not feasible due to heterogeneity, we will descriptively report the results of each study. Meta-analysis will be carried out with similar sets of confounding factors, and the results of the review will be reported according to the PRISMA guidelines.

## 29. * Analysis of subgroups or subsets.

State any planned investigation of ‘subgroups’. Be clear and specific about which type of study or participant will be included in each group or covariate investigated. State the planned analytic approach.

We will carry out a subgroup analysis by comparing MitraClip versus Non-MitraClip in patients with heart failure complicated functional mitral regurgitation.

## 30. * Type and method of review.

Select the type of review, review method and health area from the lists below.

### Type of review

Cost effectiveness

No

Diagnostic

No

Epidemiologic

No

Individual patient data (IPD) meta-analysis

No

Intervention

Yes

Living systematic review

No

Meta-analysis

Yes

Methodology

No

Narrative synthesis

No

Network meta-analysis

No

Pre-clinical

No

Prevention

No

Prognostic

No

Prospective meta-analysis (PMA)

No

Review of reviews

No

Service delivery

No

Synthesis of qualitative studies

No

Systematic review

Yes

Other

No

## Health area of the review

Alcohol/substance misuse/abuse

No

Blood and immune system

No

Cancer

No

Cardiovascular

Yes

Care of the elderly

No

Child health

No

Complementary therapies

No

COVID-19

No

Crime and justice

No

Dental

No

Digestive system

No

Ear, nose and throat

No

Education

No

Endocrine and metabolic disorders

No

Eye disorders

No

General interest

No

Genetics

No

Health inequalities/health equity

No

Infections and infestations

No

International development

No

Mental health and behavioural conditions

No

Musculoskeletal

No

Neurological

No

Nursing

No

Obstetrics and gynaecology

No

Oral health

No

Palliative care

No

Perioperative care

No

Physiotherapy

No

Pregnancy and childbirth

No

Public health (including social determinants of health)

No

Rehabilitation

No

Respiratory disorders

No

Service delivery

No

Skin disorders

No

Social care

No

Surgery

No

Tropical Medicine

No

Urological

No

Wounds, injuries and accidents

No

Violence and abuse

No

## 31. Language.

Select each language individually to add it to the list below, use the bin icon to remove any added in error.

English

There is not an English language summary

## 32. * Country.

Select the country in which the review is being carried out. For multi-national collaborations select all the countries involved.

China

## 33. Other registration details.

Name any other organisation where the systematic review title or protocol is registered (e.g. Campbell, or The Joanna Briggs Institute) together with any unique identification number assigned by them. If extracted data will be stored and made available through a repository such as the Systematic Review Data Repository (SRDR), details and a link should be included here. If none, leave blank.

## 34. Reference and/or URL for published protocol.

If the protocol for this review is published provide details (authors, title and journal details, preferably in Vancouver format)

Add web link to the published protocol.

Or, upload your published protocol here in pdf format. Note that the upload will be publicly accessible.

No I do not make this file publicly available until the review is complete

Please note that the information required in the PROSPERO registration form must be completed in full even if access to a protocol is given.

## 35. Dissemination plans.

Do you intend to publish the review on completion?

Yes

Give brief details of plans for communicating review findings.?

## 36. Keywords.

Give words or phrases that best describe the review. Separate keywords with a semicolon or new line. Keywords help PROSPERO users find your review (keywords do not appear in the public record but are included in searches). Be as specific and precise as possible. Avoid acronyms and abbreviations unless

these are in wide use.

Functional mitral regurgitation; Heart failure; MV repair; Mitral annuloplasty; MitraClip

## 37. Details of any existing review of the same topic by the same authors.

If you are registering an update of an existing review give details of the earlier versions and include a full bibliographic reference, if available.

## 38. * Current review status.

Update review status when the review is completed and when it is published.New registrations must be ongoing so this field is not editable for initial submission.

Please provide anticipated publication date

Review_Ongoing

## 39. Any additional information.

Provide any other information relevant to the registration of this review.

Indeed, there are similar reviews regarding the comparison of mitral valve replacement and mitral valve repair. However, our study consists of the most updated studies (for example, a 5-year result of the COAPT trial that was recently published); moreover, we discuss the most recent hot topic in the mitral valve repair technique, which is MitraClip. Therefore, we believe that this updated meta-analysis has some significance in clinical practice.

## 40. Details of final report/publication(s) or preprints if available.

Leave empty until publication details are available OR you have a link to a preprint (NOTE: this field is not editable for initial submission). List authors, title and journal details preferably in Vancouver format.

Give the link to the published review or preprint.
